# Multiple Variants of SARS-CoV-2 in a University Outbreak After Spring Break — Chicago, Illinois, March–May 2021

**DOI:** 10.15585/mmwr.mm7035a3

**Published:** 2021-09-03

**Authors:** Kate Doyle, Richard A. Teran, Jennita Reefhuis, Janna L. Kerins, Xueting Qiu, Stefan J. Green, Hyeree Choi, Sabrina A. Madni, Nazia Kamal, Emily Landon, Reynald Christopher Albert, Massimo Pacilli, Laura E. Furtado, Mary K. Hayden, Kevin J. Kunstman, Cindy Bethel, Lauren Megger, Marielle J. Fricchione, Isaac Ghinai

**Affiliations:** ^1^Chicago Department of Public Health, Chicago, Illinois; ^2^Epidemic Intelligence Service, CDC; ^3^Division of Birth Defects and Infant Disorders, National Center on Birth Defects and Developmental Disabilities, CDC; ^4^Rush University Medical Center, Chicago, Illinois; ^5^4ES Corporation, San Antonio, Texas; ^6^Division of Preparedness and Emerging Infections, National Center for Emerging and Zoonotic Infectious Diseases, CDC; ^7^University of Chicago, Chicago, Illinois.

To prevent transmission of SARS-CoV-2, the virus that causes COVID-19, colleges and universities have implemented multiple strategies including testing, isolation, quarantine, contact tracing, masking, and vaccination. In April 2021, the Chicago Department of Public Health (CDPH) was notified of a large cluster of students with COVID-19 at an urban university after spring break. A total of 158 cases of COVID-19 were diagnosed among undergraduate students during March 15–May 3, 2021; the majority (114; 72.2%) lived in on-campus dormitories. CDPH evaluated the role of travel and social connections, as well as the potential impact of SARS-CoV-2 variants, on transmission. Among 140 infected students who were interviewed, 89 (63.6%) reported recent travel outside Chicago during spring break, and 57 (40.7%) reported indoor social exposures. At the time of the outbreak, undergraduate-aged persons were largely ineligible for vaccination in Chicago; only three of the students with COVID-19 (1.9%) were fully vaccinated. Whole genome sequencing (WGS) of 104 specimens revealed multiple distinct SARS-CoV-2 lineages, suggesting several nearly simultaneous introductions. Most specimens (66; 63.5%) were B.1.1.222, a lineage not widely detected in Chicago before or after this outbreak. These results demonstrate the potential for COVID-19 outbreaks on university campuses after widespread student travel during breaks, at the beginning of new school terms, and when students participate in indoor social gatherings. To prevent SARS-CoV-2 transmission, colleges and universities should encourage COVID-19 vaccination; discourage unvaccinated students from travel, including during university breaks; implement serial COVID-19 screening among unvaccinated persons after university breaks; encourage masking; and implement universal serial testing for students based on community transmission levels.

## University Prevention Measures

In spring 2021, approximately 2,100 students were living on the campus of an urban university in Chicago, Illinois. In response to the COVID-19 pandemic, the university implemented numerous prevention strategies.^†^ Students living on- and off-campus were required to report positive SARS-CoV-2 test results to the university. Students living in the dormitories were required to receive testing for SARS-CoV-2 every week (serial screening); testing was offered for free by the university.^§^ During March 20–29, 2021, university activities, including classes, paused for spring break, and the university recommended that students avoid all travel during this period; dormitories remained open. After the break, students who lived on campus were advised to stay in their dormitories for 1 week, and all classes were held remotely.^¶^ In addition to regular serial screening, students who lived in dormitories were required to receive testing for SARS-CoV-2 before resuming in-person learning.

## Investigation and Response

On April 7, 2021, the university notified CDPH of 37 students with positive SARS-CoV-2 test results detected through serial screening conducted during March 29–April 5, 2021. In response to this cluster of COVID-19 cases, the university implemented a stay-at-home order for students living on campus (requiring students to stay in their dormitories), held all classes remotely, and prohibited gatherings. During the stay-at-home order, the university modified the screening schedule to require testing for students living on campus twice during the first 10 days of the order. In consultation with CDPH, after additional testing found few cases, the university lifted the order after 14 days.

A case was defined as receipt of a positive SARS-CoV-2 test result by an undergraduate student living on or near the university campus during March 15–May 18, 2021.** For all students with COVID-19, the university provided information on residence (on-campus dormitory or off-campus), age, gender, and positive specimen collection date. CDPH conducted interviews to collect information on demographic characteristics, clinical signs or symptoms, travel history, social activities, attendance at social gatherings, and close contacts. Diagnostic testing history and results were extracted from state surveillance and vaccination records from immunization registry systems. Available specimens were sequenced and assigned a lineage.^††^ Similar sequences (differing by fewer than five nucleotides) were assumed to represent a single viral introduction.^§§^ To identify possible geographic sources of importations, outbreak lineages were compared with all contemporaneous sequences of the same lineage available on the Global Initiative on Sharing All Influenza Data (GISAID) platform. Descriptive and social network analyses were completed using R (version 4.1.0; R Foundation) and MicrobeTrace (version 0.7.0; CDC), respectively. This activity was reviewed by CDC and was conducted consistent with applicable federal law and CDC policy.^¶¶^

**FIGURE 1:**
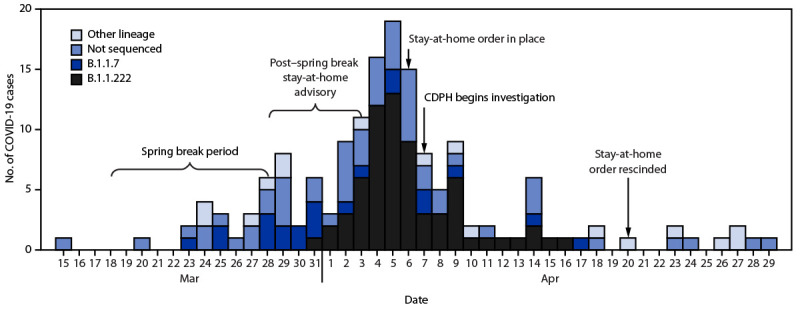
Date of onset* and viral lineage among undergraduate students with COVID-19 (n = 158) — Chicago, Illinois, March–April 2021 **Abbreviation:** CDPH = Chicago Department of Public Health. * Or date of specimen collection for asymptomatic and presymptomatic persons. One specimen was collected and tested in May, but the date of symptom onset for the student was in April.

A total of 158 COVID-19 cases were identified among undergraduate students (Figure 1), including 76 (48.1%) in women; the median age of students with COVID-19 was 19.4 years (interquartile range = 18.9–20.3 years) (Table). A total of 114 (72.2%) students with COVID-19 lived in dormitories (Supplementary Table, https://stacks.cdc.gov/view/cdc/109260); the rest lived off-campus but near the university.

**TABLE:** Characteristics of undergraduate students with COVID-19 (n = 158) — Chicago, Illinois, March–May 2021

Characteristic (no. with available information)	No. (%)
**Demographics (158)**	
Female	76 (48.1)
Median age, yrs (IQR)	19.4 (18.9–20.2)
**Residence (158)**	
Dormitory A	35 (22.2)
Dormitory B	32 (20.3)
Dormitory C	31 (19.6)
Dormitory D	7 (4.4)
Dormitory E	5 (3.2)
Dormitory F	4 (2.5)
Off-campus	44 (27.8)
Previous COVID-19 diagnosis >90 days before test date*	1 (0.6)
**Interviewed (140)**	
Symptomatic	127 (90.7)
Provided at least one contact name	88 (62.9)
Reported indoor social exposure	57 (40.7)
Reported party exposure	3 (2.1)
Reported travel	89 (63.6)
**Vaccination (140)**	
Reported not vaccinated	93 (66.4)
Reported partially vaccinated	43 (30.7)
Reported fully vaccinated^†^	3 (2.1)
**Travel destinations (89)**	
Florida	20 (22.5)
California	11 (12.4)
New York	11 (12.4)
Colorado	5 (5.6)
Within Illinois	3 (3.4)
Other U.S. states	32 (36.0)
International	6 (6.7)
**Purpose of travel (89)**	
Vacation away from home	43 (48.3)
Visiting home	23 (25.8)
Moving to campus	3 (3.4)
Unknown	20 (22.5)
**Lineage (104)**	
B.1.1.222	66 (63.5)
B.1.1.7	22 (21.2)
P.1	9 (8.7)
B.1.526	3 (2.9)
B.1.526.1	1 (1.0)
B.1.526.2	1 (1.0)
B.1.1	1 (1.0)
B.1.429	1 (1.0)

**Abbreviation:** IQR = interquartile range.

* Previous diagnosis of COVID-19 was laboratory-confirmed. † Vaccination information was collected by self-report and verified, when possible, with the state immunization registry. The three persons who reported full vaccination could not be verified because vaccinations were administered out of state (two) and as part of a clinical trial (one).

Among the 158 students with COVID-19, 140 (88.6%) were interviewed, among whom 127 (90.7%) reported at least one COVID-19 symptom (Table). Two were evaluated in an emergency department after diagnosis; no infected student was hospitalized or died. One student with COVID-19 had a previous laboratory-confirmed diagnosis of COVID-19 >90 days before the infection was identified during the investigation period. Among all interviewed students with COVID-19, 93 (66.4%) were unvaccinated, and 43 (30.7%) were partially vaccinated (i.e., received 1 dose of a 2-dose COVID-19 vaccine series or completed a vaccine series <14 days before diagnosis). Three (1.9%) students with COVID-19 reported being fully vaccinated; two of these students experienced symptoms. 

**FIGURE 2:**
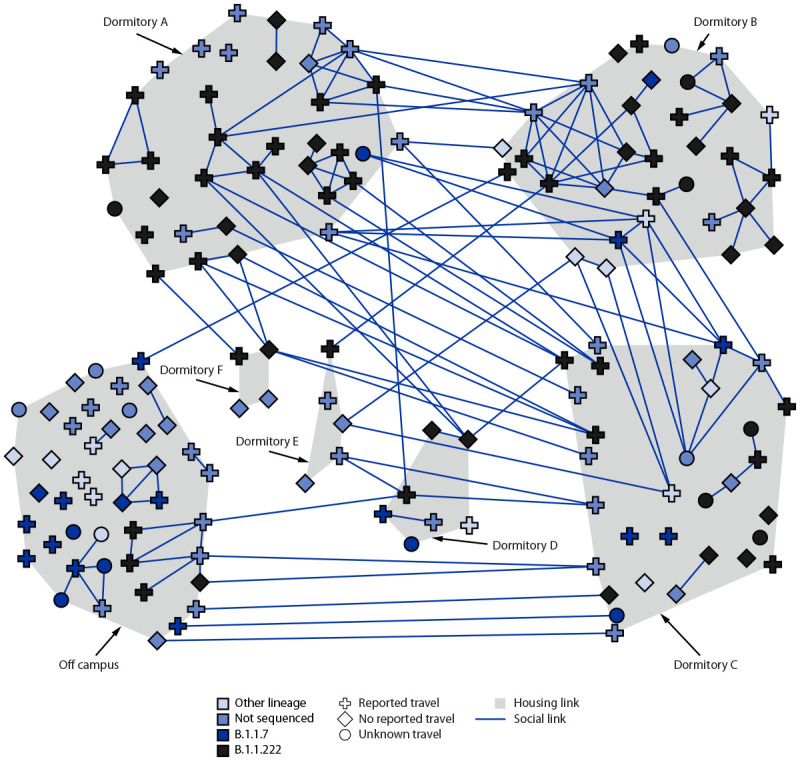
Social networks among undergraduate students with COVID-19 (n = 158), by residence and viral lineage — Chicago, Illinois, March–May 2021

The majority (88; 62.9%) of students with COVID-19 provided the name of at least one other student with COVID-19 with whom they had had contact in the 2 weeks preceding symptom onset or test date. Fifty-seven (40.7%) students with COVID-19 described unmasked indoor exposures to other students at small gatherings, meals, or while studying. Although the university was aware of several large gatherings, only three infected students (2.1%) reported having attended a party. A network diagram was constructed to show social connections, residence, travel, and viral lineage (Figure 2). Based on interview data, 25 groups of socially connected students with COVID-19 (clusters) were identified; the median cluster size was two, and the maximum was 45. Several social groups included multiple dormitories.

Overall, 89 (63.6%) interviewed students with COVID-19 reported travel outside Chicago during spring break. Fourteen students traveled with at least one other infected student in five different travel groups. Destinations included seven different countries and 23 U.S. states; the most commonly visited states were California, Colorado, Florida, and New York (Table). The most commonly reported reason for travel was vacation (43; 48.3%).

Residual specimens were available for 120 (75.9%) infected students, 104 (86.7%) of which were successfully sequenced. Sequences were assigned nine different lineages, mostly B.1.1.222 (66; 63.5%), followed by B.1.1.7 (Alpha) (22; 21.2%) (Table) (Supplementary Figure, https://stacks.cdc.gov/view/cdc/109259). All B.1.1.222 sequences differed by fewer than five nucleotides and likely represent a single source introduction. When compared with sequences in GISAID, the B.1.1.222 sequences in this outbreak were most closely related to specimens from California. Eight of the 66 students (12.1%) infected with the SARS-CoV-2 B.1.1.222 lineage had a history of travel to California. In Dormitory A, 25 of 35 (71.4%) specimens from infected students were sequenced, and 24 of 25 (96.0%) were confirmed as the B.1.1.222 lineage (Figure 2). Among B.1.1.7 (Alpha) specimens, 10 groups of sequences differed by five or more nucleotides, indicating multiple separate importations; groups ranged in size from one to five students. B.1.1.7 was circulating widely in Chicago and elsewhere in the United States at the time of this outbreak. Among the students who traveled together, some travel groups had the same lineage (though students reported additional close contact on campus), while other groups included several lineages. Specimens from all three fully vaccinated students were available; however, only one (from a symptomatic student) was successfully sequenced as a B.1.1.222 lineage. 

## Discussion

Previous reports have described outbreaks of COVID-19 among university students with complex social networks and social exposures (*1*–*5*). In this outbreak, 158 cases of COVID-19 were identified after many unvaccinated students traveled during a university break, despite university policies advising against travel. Subsequent on-campus gatherings led to further transmission within and across social networks, including between dormitories. Notably, this outbreak occurred immediately before expansion of eligibility for vaccination in Chicago; undergraduate-aged persons were largely ineligible for vaccination before April 19, 2021.***

WGS identified several lineages and multiple distinct introductions of SARS-CoV-2 that were possibly driven by student travel. Phylogenetic analyses illustrated gaps in the social network; for example, several students with no reported social connections were infected with nearly identical strains of B.1.1.222, a lineage not widely identified in Chicago before or after this outbreak.^†††^ Transmission likely occurred among students without known social connections or through undetected cases associated with the outbreak, although these links cannot be confirmed with available case interview data.

The findings in this report are subject to at least four limitations. First, some students with COVID-19 refused interviews, omitted critical details, or provided false and conflicting information, such as denying travel when other students indicated that they had traveled together. This reticence limited the ability to thoroughly assess social networks and transmission chains. Second, serial screening was mandatory only for students living on-campus; students living off-campus might have had COVID-19 but did not receive testing during the outbreak period. Given potentially undiagnosed infections, the magnitude of the outbreak might have been greater than described. Third, not all SARS-CoV-2 specimens could be sequenced; additional viral introductions or transmission chains might have been missed. Finally, because publicly available sequence data include only a subset of all viruses, the source of viral introductions could not be definitively identified.

These findings support existing CDC recommendations for the control of COVID-19 in colleges and universities; these recommendations are especially important given the rapid spread of the B.1.617.2 (Delta) variant of concern.^§§§^ Serial testing successfully detected an outbreak among university undergraduates; isolation of students with COVID-19, contact tracing, and university-wide prevention measures contributed to reductions in transmission. Nevertheless, unvaccinated persons traveling during a university break and subsequent socializing among students resulted in multiple clusters of COVID-19 before vaccines were widely offered to undergraduate-aged persons in Chicago. Vaccination is the leading prevention strategy to protect persons from COVID-19, and colleges and universities can benefit from encouraging vaccination for all students, faculty, and staff members. In settings where not everyone is fully vaccinated or where students have contact with community members who are not fully vaccinated, colleges and universities can encourage unvaccinated students to refrain from travel; implement serial screening testing for unvaccinated students, faculty, and staff members after university breaks; test for SARS-CoV-2 based on community transmission levels; encourage masking indoors; and make free, voluntary testing readily available, including for fully vaccinated persons who are experiencing COVID-19 symptoms.^¶¶¶^

**Summary**

**What is already known about this topic?**

SARS-CoV-2 transmission on college and university campuses can occur when unvaccinated students return to campus after travel or attend social gatherings.

**What is added by this report?**

After spring break 2021, COVID-19 cases increased rapidly at a Chicago university despite mitigation measures. Interviews indicated that the majority of cases occurred in unvaccinated persons with a history of recent travel. Sequencing corroborated multiple introductions to campus and demonstrated that even a single importation can result in many cases.

**What are the implications for public health practice?**

To mitigate SARS-CoV-2 transmission, colleges and universities can encourage COVID-19 vaccination; discourage unvaccinated students from traveling, including during university breaks; implement serial screening after university breaks; test based on community transmission; and encourage masking.

## Footnotes

* These authors contributed equally to this report.

† For the 2020–21 academic year, dormitory capacities were reduced, bedrooms were single-occupancy, and numerous changes to physical infrastructure were made. All students were required to sign a health agreement and pledge to avoid social gatherings. The university maintained separate housing space for students with positive SARS-CoV-2 test results; these students were required to follow isolation procedures.

§ Real-time reverse transcription–polymerase chain reaction testing was performed on anterior nasal swab specimens collected by trained medical assistants.

¶ Students were permitted to leave their dormitories to take walks, buy food, or pick up meals from the dining halls.

** The investigation period began 2 weeks before the start of spring break and ended 2 weeks after the last positive SARS-CoV-2 test result for an undergraduate student. Any positive test result among undergraduate students who lived on campus or in Chicago was evaluated for inclusion. Graduate students and staff members with COVID-19 were not included in this outbreak case definition. Assessment of cases among these populations indicated very limited or no interaction with undergraduate students who lived on or very near campus.

†† Available SARS-CoV-2 positive specimens were submitted to CDPH's Regional Innovative Public Health Laboratory for whole genome sequencing using the Swift Amplicon SARS-CoV-2 Panel (Swift Biosciences) and deep sequencing on an Illumina instrument. Viral lineages were assigned using the Phylogenetic Assignment of Named Global Outbreak Lineages (PANGOLIN) tool (version v3.0.2; Rambaut Laboratory). Nucleotide differences were assessed with IQ-TREE.

§§ Among the specimens of B.1.1.222 lineage, the majority of sequences were genetically similar (within two nucleotides). Although there is no standard genomic definition of sequences linked by transmission, this level of similarity, together with the lack of detection of similar contemporaneous specimens in Chicago, is likely consistent with one viral introduction.

¶¶ 45 C.F.R. part 46.102(l)(2), 21 C.F.R. part 56; 42 U.S.C. Sect. 241(d); 5 U.S.C. Sect. 552a; 44 U.S.C. Sect. 3501 et seq.

***
https://www.chicago.gov/city/en/sites/covid19-vaccine/home/vaccine-distribution-phases.html

††† Only one other B.1.1.222 sequence from a specimen collected in 2021 in Chicago was available on GISAID (out of 674 Chicago sequences from 2021 not associated with this outbreak). This sequence, from a specimen collected 3 months before this outbreak, differed by more than five nucleotides from the largely homogenous sequences observed in this outbreak.

§§§
https://www.cdc.gov/coronavirus/2019-ncov/community/colleges-universities/considerations.html

¶¶¶
https://www.cdc.gov/coronavirus/2019-ncov/community/colleges-universities/considerations.html#section3
